# Ultra-High-Field fMRI Reveals a Role for the Subiculum in Scene Perceptual Discrimination

**DOI:** 10.1523/JNEUROSCI.3225-16.2017

**Published:** 2017-03-22

**Authors:** Carl J. Hodgetts, Natalie L. Voets, Adam G. Thomas, Stuart Clare, Andrew D. Lawrence, Kim S. Graham

**Affiliations:** ^1^Cardiff University Brain Research Imaging Centre, School of Psychology, Cardiff University, Cardiff CF24 4HQ, United Kingdom,; ^2^Oxford Centre for Functional MRI of the Brain, Nuffield Department of Clinical Neurosciences, University of Oxford, John Radcliffe Hospital, Oxford OX3 9DU, United Kingdom, and; ^3^Section on Functional Imaging Methods, NIMH, National Institutes of Health, Bethesda, Maryland 20892-1148

**Keywords:** 7 T fMRI, episodic memory, hippocampus, medial temporal lobe, perception, scene processing

## Abstract

Recent “representational” accounts suggest a key role for the hippocampus in complex scene perception. Due to limitations in scanner field strength, however, the functional neuroanatomy of hippocampal-dependent scene perception is unknown. Here, we applied 7 T high-resolution functional magnetic resonance imaging (fMRI) alongside a perceptual oddity task, modified from nonhuman primate studies. This task requires subjects to discriminate highly similar scenes, faces, or objects from multiple viewpoints, and has revealed selective impairments during scene discrimination following hippocampal lesions. Region-of-interest analyses identified a preferential response in the subiculum subfield of the hippocampus during scene, but not face or object, discriminations. Notably, this effect was in the anteromedial subiculum and was not modulated by whether scenes were subsequently remembered or forgotten. These results highlight the value of ultra-high-field fMRI in generating more refined, anatomically informed, functional accounts of hippocampal contributions to cognition, and a unique role for the human subiculum in discrimination of complex scenes from different viewpoints.

**SIGNIFICANCE STATEMENT** There is increasing evidence that the human hippocampus supports functions beyond just episodic memory, with human lesion studies suggesting a contribution to the perceptual processing of navigationally relevant, complex scenes. While the hippocampus itself contains several small, functionally distinct subfields, examining the role of these in scene processing has been previously limited by scanner field strength. By applying ultra-high-resolution 7 T fMRI, we delineated the functional contribution of individual hippocampal subfields during a perceptual discrimination task for scenes, faces, and objects. This demonstrated that the discrimination of scenes, relative to faces and objects, recruits the anterior subicular region of the hippocampus, regardless of whether scenes were subsequently remembered or forgotten.

## Introduction

The hippocampus has long been considered an exclusive declarative memory system (Squire and Dede, 2015). Mounting evidence suggests, however, that this structure additionally supports nonmnemonic functions, such as complex scene perception (Gaffan, 2002; Graham et al., 2010). Hippocampal amnesics, for instance, are impaired during scene discriminations, particularly when scenes are presented from different viewpoints (Lee et al., 2005a). This has led to the view that the hippocampus supports scene processing if the task at hand requires the formation of flexible conjunctions of features constituting a visual scene, be it during long-term memory (Taylor et al., 2007; Bird et al., 2008), working memory (Hannula et al., 2006; Lee and Rudebeck, 2010; Zeidman et al., 2015a), or perception (Lee et al., 2005a; Aly et al., 2013). This putative function may contribute to the generation of map-like allocentric representations (Fidalgo and Martin, 2016), and underpin the broader role of the hippocampus in episodic memory via re-experiencing spatial context (Burgess et al., 2002; Schiller et al., 2015).

Despite strong evidence for the representational view outlined above (Graham et al., 2010), it is not without criticism (Suzuki and Baxter, 2009). While several neuropsychological studies have observed scene-related impairments following hippocampal damage (Lee et al., 2005a; for review, see Graham et al., 2010; Mullally et al., 2012; Maguire et al., 2016), others have failed to replicate these effects (Squire et al., 2006; Kim et al., 2015). While methodological differences may account for these discrepancies (Suzuki, 2009; Maguire et al., 2016), there is also limited understanding of how patterns of hippocampal subregional atrophy might lead to these deficits. Critically, the hippocampus is not a single unitary brain structure but contains four cellularly and functionally distinct subfields [CA1, CA2/3, dentate gyrus (DG), and subiculum], with functional gradients posited along both its long (Poppenk et al., 2013; Strange et al., 2014; anterior–posterior) and transverse (medial–lateral) axes (Henriksen et al., 2010; Maass et al., 2015). Functionally delineating these subregions is challenging, however, due to the predominant use of scanners with field strengths of 1.5–3 T, which has limited high-resolution studies of hippocampal scene processing to functional in-plane resolutions of ∼1.5–2 mm (Diana et al., 2008; Preston et al., 2010; Bonnici et al., 2012; Zeidman et al., 2015b). These constraints on in/out-of-plane resolution (both functionally and structurally) makes it particularly difficult to characterize subregions displaying high intraslice and interslice variation in subfield morphology/organization, such as the anterior hippocampus (Zeidman and Maguire, 2016).

Indeed, while several 3 T fMRI studies report scene sensitivity in the posterior hippocampus (Lee et al., 2008; Barense et al., 2010; Liang et al., 2013), there is emerging evidence that the anterior hippocampus may be particularly responsive to scenes (Lee et al., 2013; Hodgetts et al., 2016; Zeidman and Maguire, 2016), potentially via its broader functional connectivity within an anterior scene processing network (Baldassano et al., 2016). A recent account proposes a role for the anterior hippocampus—and in particular the subiculum—in constructing internal scene “models” (Zeidman and Maguire, 2016). Such representations may be particularly relevant during perception if scenes must be compared across viewpoints, as this involves integration of scene perspectives into a coherent view-invariant representation (Graham et al., 2010). Thus, it is possible that such tasks will preferentially engage substructures that predominate the anterior hippocampus, such as the subiculum.

**Figure 1.**
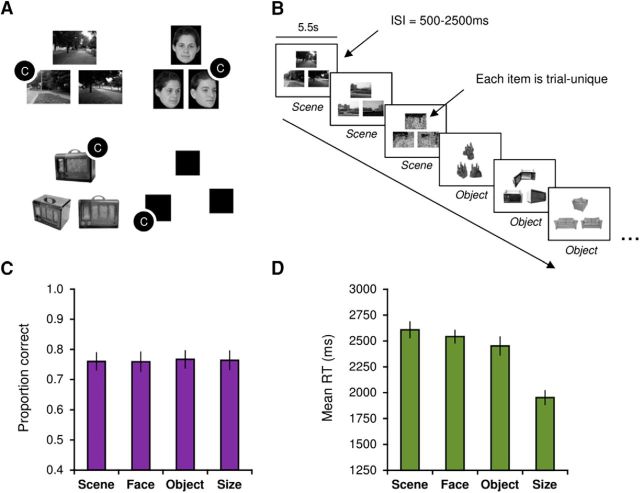
***A***, Examples of scene, face, object, and size (baseline) oddity trials (C markers indicate correct odd-one-out responses, which were selected using a button box in the scanner). Faces were obtained from the Psychological Image Collection at Stirling (http://pics.stir.ac.uk/); objects were taken from the Hemera Photo-Objects 50,000, Volumes 1–3. ***B***, Schematic illustration of the oddity task. Trials for each category were presented in miniblocks of three trials (shown in the figure for scenes and objects). Trials were presented for 5500 ms with a jittered intertrial interval of 500–2500 ms. ***C***, Accuracy data (proportion correct) for the oddity task. ***D***, RT data for the oddity task. Error bars represent ± SE.

To functionally distinguish hippocampal subfields during scene perception, we applied high-field 7 T MRI with a functional resolution of 1.2 mm isotropic (corresponding to 1.72 mm^3^). Ultra-high-resolution T2*-weighted images were also acquired at a resolution of 0.6 mm isotropic (corresponding to 0.216 mm^3^), allowing the convoluted internal structure of the hippocampus to be visualized along its long axis (Wisse et al., 2012). To draw correspondence with lesion studies across species (Buckley et al., 2001), perceptual processing was probed using an “oddity” paradigm in which subjects make odd-one-out decisions between highly similar scenes, faces, or objects, presented across multiple viewpoints. A trial-unique approach is typically used where stimuli to-be-discriminated are never repeated, once shown, in the task, and stimuli are presented concurrently to ensure no delay across items (Lee et al., 2005a; Behrmann et al., 2016; Fig. 1*A*,*B*). As such, this task provides a strong test of hippocampal subfield contributions to nonmnemonic processing (Yonelinas, 2013).

## Materials and Methods

### 

#### 

##### Subjects.

Twenty-five healthy volunteers, with no history of neurological or psychiatric illness, were recruited from the University of Oxford and Oxford Brookes University (9 male; aged 18–35 years; mean age, 25 years; SD, 4 years). All subjects were fluent English speakers with normal or corrected-to-normal vision. All subjects provided written informed consent before taking part. The research project was approved by the University of Oxford research ethics committee.

##### Task and design.

During the oddity task, subjects were presented with three stimuli on each trial (top center; bottom left; bottom right) and instructed to select the odd one out as quickly and as accurately as possible (Fig. 1*A*). The scene stimuli were real-world, greyscale photographs of outdoor environments. On each trial, subjects viewed two images of a single location from different viewpoints and one different location. Face stimuli were greyscale photographs of human faces, half of which were male, and were obtained from the Psychological Image Collection at Stirling (http://pics.stir.ac.uk/). Individual faces were overlaid on a black background (170 × 216 pixels). Two faces were the same individual presented from different viewpoints (or with different facial expression) and the target was a different face presented from a different viewpoint. Objects were taken from the Hemera Photo-Objects 50,000, Volumes 1–3. As above, two objects were the same from different viewpoints, and the third (target) was a highly similar object from the same subordinate-level object category. For the “size” task, three black squares were presented. The position of the squares on the screen was jittered so that none of the edges lined up along vertical or horizontal axes. On each trial, two of the squares were identical in size and a third square was either slightly larger or smaller. The difference in length between target and nontargets could vary between 9 and 15 pixels. All stimuli were trial-unique (i.e., never repeated in the task). Subjects were shown a practice trial for each category before going into the scanner and indicated to the experimenter their response.

Stimuli were presented in the scanner using Presentation (Neurobehavioural Systems) and projected onto the screen behind the subject using an Eiki LC-XL100 projector system (resolution, 1024 × 768; refresh rate: 60 Hz). Button responses in the scanner were acquired using a right-hand MR-compatible button box. Each trial was presented for 5500 ms with a jittered intertrial interval of 500–2500 ms (Fig. 1*B*). The task was administered in the scanner over three fMRI runs. Within each run, trials for a given condition (scene, face, object, and “size” baseline) were presented in miniblocks of three successive trials. The order in which category miniblocks were presented was counterbalanced across subjects. Overall, 15 trials were presented per category per run, resulting in 45 trials per condition overall. An equal number of targets appeared at each screen position (i.e., top center; bottom left; bottom right) within each stimulus condition. Outside the scanner, subjects completed a surprise recognition memory task. The 45 target items (“old”), alongside 45 foils (“new”), for each category were presented in the center of the display. The order of stimuli (across targets and foils) was fully randomized.

##### MRI data acquisition.

Data were acquired using a Siemens 7 T Magnetom system, in combination with a 32-channel head coil (Nova Medical). Whole-head T1-weighted images were acquired with an MPRAGE sequence at 1 × 1 × 1 mm (TE = 2.82 ms; TR = 2200 ms; flip angle, 7°). Blood oxygen level-dependent (BOLD) fMRI data were acquired using a T2*-weighted echo planar imaging (EPI) sequence. The oddity task was presented across three fMRI runs, each consisting of 212 volumes and lasting ∼7 min each (30 slices; TE = 25 ms, TR = 2000 ms; voxel size, 1.2 × 1.2 × 1.2 mm; partial field-of-view, 192 mm; partial Fourier, 6/8; parallel imaging with GRAPPA factor, 2; bandwidth, 1562 Hz/pixel; echo spacing, 0.72 ms; flip angle, 90°). Slices were oriented parallel to the hippocampal long axis and acquired in a descending interleaved (odd–even) order. Three volumes were discarded at the start of each run to allow for magnetization equilibrium. To aid the coregistration of partial field-of-view images, an additional whole-brain T2*-weighted EPI volume was collected using identical image parameters. A field map was acquired (using the same slice orientation as the functional acquisition) to improve registration and reduce image distortion from magnetic-field inhomogeneity (TE 1 = 4.08 ms; TE 2 = 5.1 ms; TR = 620 ms; field-of-view, 192 mm; flip angle, 39°). Two T2*-weighted ultra-high-resolution structural images were acquired in opposing phase-encoding directions (left-to-right; right-to-left; 44 slices; TE = 25.7 ms; TR = 50 ms; voxel size, 0.6 × 0.6 × 0.6 mm; partial Fourier, 6/8; field-of-view, 192 mm). Slices were aligned orthogonal to the hippocampal main axis based on visual inspection by the experimenters and radiographer.

##### MRI preprocessing.

Functional MRI data were preprocessed using the Functional MRI of the Brain (FMRIB) Software Library (FSL; Jenkinson et al., 2012). Following conversion of raw image data to NifTI, T1-weighted images were stripped of nonbrain tissue using the BET [Brain Extraction Tool (Smith, 2002)] and bias field corrected using FAST [FMRIB's Automatic Segmentation Tool (Zhang et al., 2001)]. BOLD fMRI preprocessing and analysis was performed using the FMRI Expert Analysis Tool (FEAT) Version 6. Analysis of functional data included the following preprocessing stages: motion correction using MCFLIRT [Motion Correction tool based on techniques used in FMRIB's Linear Image Registration Tool (FLIRT); Jenkinson et al., 2002]; high-pass temporal filtering (Gaussian-weighted least-squares straight line fitting, with σ = 50 s); and field map unwarping of EPI data using Fugue (tools for EPI distortion correction; Jenkinson et al., 2002). For group-level analyses, we applied a Gaussian kernel of full-width half-maximum (FWHM) 2 mm. No smoothing was applied for the individual-level subfield region-of-interest (ROI) analysis. Time-series statistical analysis was performed using FMRIB's Improved Linear Model with local autocorrelation correction (Woolrich et al., 2001). Registration of functional images to T1-weighted MPRAGE images (per subject) involved the concatenation of the following two transforms: (1) registration of partial field-of-view functional images to whole-brain EPI images using FLIRT (degrees-of-freedom, 3), and (2) registration of whole-brain EPI images to the T1-weighted structural scan using epi_reg, which uses white–gray matter contrast information to nonlinearly register EPI images to T1-weighted images. Nonlinear registration of the functional data to the Montreal Neurological Institute (MNI152) 1 mm template (for group averaging) was performed using FNIRT (FMRIB's Nonlinear Image Registration Tool). The BOLD signal was modeled using a double-gamma hemodynamic response function. Coordinates of significant group-level effects are reported in MNI space.

##### Subject exclusion.

No subject displayed head movement of >1 EPI voxel (1.2 mm). One subject was removed from the analysis due to an incidental finding on their MRI and another participant because of excessive susceptibility artifacts in the anterior temporal lobe precluding accurate hippocampal segmentation. A total of 23 subjects were included in all subsequent analyses.

##### fMRI data analysis.

Four explanatory variables, comprising correct scene, face, object, and size oddity judgements, were used to model the time-course data at the individual-subject level. A general linear model (GLM) was implemented within each fMRI run to examine the BOLD response associated with the four main predictors. An additional confound matrix was added to the GLM to account for volume-wise nonlinear motion effects using FSL Motion Outliers. A parameter estimate image was created for each explanatory variable against active baseline (size oddity) and for several planned contrasts to examine differences in activity across our three key categories of interest: (1) the main effect of scenes: scenes > faces + objects; (2) the main effect of faces: faces > scenes + objects; and (3) the main effect of objects: objects > scenes + faces. The three individual runs for each subject were combined using a fixed-effects model in FEAT. Group-level analyses were performed using the FMRIB Local Analysis of Mixed Effects tool version 1 (FLAME 1; Beckmann et al., 2003; Woolrich et al., 2004). For the group-level analyses, the resulting group-averaged statistical maps were thresholded with a cluster-determining threshold of *p* = 0.0001 (Eklund et al., 2016) with a familywise error-corrected cluster threshold of *p* < 0.05 based on Gaussian random fields theory. The hippocampal ROI at the group level incorporated bilateral hippocampus probabilistic labels from the Harvard–Oxford subcortical atlas (thresholded at 50%) and the subiculum from the Jülich histological atlas (Amunts et al., 2005). The subiculum was thresholded at 75% to constrain the ROI to gray matter. Inferences relating to the subfield location of significant group-level voxels were made using the probabilistic cytoarchitectonic maps from the Jülich histological atlas.

##### Segmentation of hippocampus.

To perform our subject-specific ROI analysis, subfield ROIs were segmented manually on individual subjects' ultra-high-resolution T2*-weighted images (0.6 mm isotropic) using ITK-SNAP (www.itksnap.org). To increase signal-to-noise in these images, the two T2*-weighted images (see MRI data acquisition) were coaligned and averaged for each subject.

**Figure 2.**
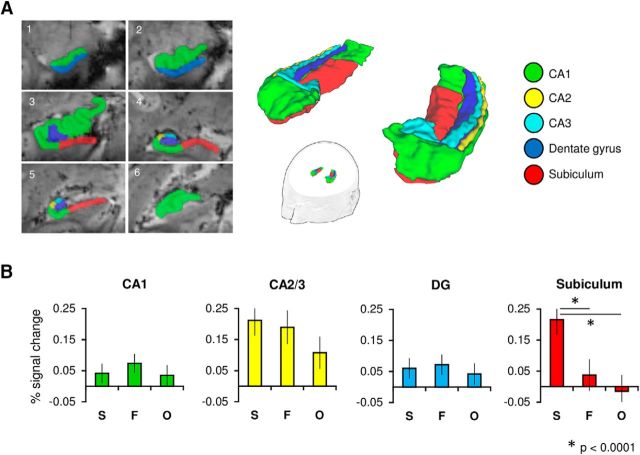
***A***, Hippocampal subfields (CA1, CA2, CA3, DG, and subiculum) were manually segmented on subjects' ultra-high-resolution T2*-weighted images. Six representative coronal slices of an individual subject's segmentation are shown (left hemisphere; 1, anterior; 6, posterior). Regions CA2 and CA3 were later concatenated as their small size precluded accurate functional localization at our coarser functional resolution of 1.2 mm isotropic. ***B***, Mean percentage signal change plots for correct scene (S), face (F), and object (O) judgements (relative to size baseline) for each hippocampal subfield ROI. Error bars represent ± SE.

Based on a published 7 T protocol (Wisse et al., 2012, 2014), the hippocampus was subdivided into five subfields (CA1, CA2, CA3, DG, subiculum), with additional reference to previous literature (Duvernoy, 1988; Mueller et al., 2007; Winterburn et al., 2013). This protocol has been previously validated in volumetric studies (Wisse et al., 2014) and has been subject to detailed evaluation against several protocols in the literature (Yushkevich et al., 2015). Slices from this segmentation procedure are shown in Figure 2*A*. Segmentation began on the most anterior slice of the hippocampal head (Wisse et al., 2012). Here, the part superior to the uncal sulcus was labeled CA1 and the superior part as the subiculum; this was continued until the emergence of the DG. At this point, the subiculum was laterally bordered by the CA1 at the medial point of the DG. This border was defined by a line perpendicular to the subicular long axis. This CA1/subiculum border was maintained—independent of variation in the location of the DG—until the uncus was no longer visible (i.e., in the hippocampal head). Note, the first slice in which the uncus was no longer visible also defined the border between the anterior and posterior hippocampus (see Results; Poppenk et al., 2013). At this point, the subiculum was again laterally bordered by CA1 at the most medial point of the DG. A line at this point defined the CA1/subiculum border throughout the body and tail until the subiculum was no longer present. When the uncal sulcus could be traced to the medial surface, the subiculum border was defined by drawing a line between the most medial point of the gray matter and the most medial point of the white matter. This reflected the boundary between the hippocampus and the entorhinal cortex (not segmented) in anterior slices, and between the hippocampus and parahippocampal cortex in more posterior slices. This border continued throughout the hippocampus, until the subiculum was no longer segmented in the hippocampal tail, at which point this became the most medial point of CA1. The tail was defined as the first slice on which the wing of the ambient cistern appears (Yushkevich et al., 2010). The subiculum was segmented in the tail until the initial appearance of the anterior calcarine sulcus.

Segmentation of CA2 and CA3 was initiated two slices anterior to the point where the uncus separates from the hippocampus. At this point, a line drawn from the lateral DG to the superior hippocampus (perpendicular to the horizontal hippocampal axis) defined the CA1/CA2 boundary. The CA2/CA3 border was defined as the medial side of a virtual square (Wisse et al., 2012), situated with its lateral side contacting the CA1/CA2 border. This border was used in subsequent slices until CA2 and CA3 were no longer segmented in the hippocampal tail. This was the point in which the superior part of the hippocampus fully encloses the DG (Winterburn et al., 2013). At this point, the whole CA layer was labeled as CA1. Segmentation of the hippocampus ended two slices posterior to this point. The uncus was labeled as CA3 at the point at which it separates from the hippocampus. The division between the CA fields and the DG was defined as the continuation of the hypointense line representing stratum lacunosum-moleculare of CA and the molecular layer of the DG.

##### Subfield ROI analysis.

Following segmentation of T2*-weighted images, subfield ROIs were registered to each subject's MPRAGE for further verification. These MPRAGE segmentations were visually inspected in detail and, if needed, amended. Finally, ROIs were registered to the individual mean functional EPI images (one for each fMRI run) using FLIRT and percentage signal change values extracted for each oddity condition (vs size oddity baseline). By registering each subject's high-resolution T2*-weighted hippocampal segmentations to the individual-level functional images (1.2 mm isotropic), this analysis afforded high anatomical specificity relative to the group-level approach. Percentage signal change values were extracted by scaling the parameter estimates for each contrast by the baseline-to-maximum range of an isolated 5 s event (see http://mumford.bol.ucla.edu/perchange_guide.pdf). To derive a single value for each subfield, percentage signal change values (averaged across voxels within each ROI) were averaged across the three runs collected for each subject. These values were compared using a within-subjects Greenhouse–Geisser-corrected (Greenhouse and Geisser, 1959) ANOVA in SPSS Statistics 23 (IBM).

## Results

### Behavioral results

Subjects accurately performed the task and showed above-chance task accuracy in all task conditions (>33%). One-way ANOVA (Greenhouse–Geisser-corrected) revealed no significant effect of stimulus category on task accuracy (*F*_(3,66)_ = 0.1, *p* = 0.94, η^2^_p_ = 0.02; Fig. 1*C*). For correct trial response time (RT), there was a significant main effect of stimulus category (*F*_(3,66)_ = 52.66, *p* < 0.001, η^2^_p_ = 0.71; Fig. 1*D*). Two-tailed paired sample *t* tests, Bonferroni-corrected for multiple comparisons (α = 0.05/6 = 0.008), found that RTs were significantly faster during correct size relative to scene (*p* < 0.001), face (*p* < 0.001), and object (*p* < 0.001) trials. A significant difference was also observed between scene and object correct trial RTs (*p* < 0.01; Table 1).

**Table 1.** Behavioral performance for the perceptual oddity task

Category	Mean	SD	SE
Accuracy (proportion correct)			
Scene	0.76	0.14	0.03
Face	0.76	0.14	0.03
Object	0.77	0.16	0.03
Size	0.76	0.15	0.03
RT (ms)			
Scene	2608	421	88
Face	2543	369	77
Object	2452	294	61
Size	1952	327	68

### Comparing subfield BOLD response during perceptual oddity

BOLD signal change during correct scene, face, and object oddity trials (relative to size baseline) was compared within our manually defined subfield ROIs (CA1, CA2/3, DG, subiculum; Fig. 2*A*). We found that the effect of stimulus category on hippocampal BOLD response was found to differ across subfield ROIs (*F*_(6,132)_ = 7.2, *p* < 0.001, η^2^_p_ = 0.25). A significant difference between oddity conditions was found in the subiculum (*F*_(2,44)_ = 14.14, *p* < 0.001, η^2^_p_ = 0.39; Fig. 2*B*). As shown in Figure 2*B*, the response in subiculum for correct scene oddity judgements was found to be significantly greater than both faces (*p* < 0.001) and objects (*p* < 0.001), whereas the subicular response during correct face and object oddity trials did not differ significantly (*p* = 0.27). There were no significant differences between the fMRI signal response for correct scene, face, and object oddity trials in either CA1 (*p* = 0.37) or the DG (*p* = 0.58), with only a trend in CA2/3 (*p* = 0.06). One-sample *t* tests were conducted for each condition against the size baseline (Bonferroni-corrected *p* = 0.05/3 = 0.017). These revealed significantly greater activity for scenes (*p* < 0.001) and faces (*p* = 0.002), but not objects (*p* = 0.046).

To provide evidence that the observed effects (i.e., increased BOLD response specifically for scenes in the subiculum) reflect differences in perceptual processing rather than long-term memory encoding (Barense et al., 2011; Lee et al., 2013), we performed an additional analysis comparing category-wise BOLD response for target items subsequently remembered versus forgotten in a surprise recognition memory test. Six participants were removed from this analysis due to poor memory performance (proportion of hits − proportion of false alarms <0.1 in any task condition) resulting in a sample of *n* = 17. In the subiculum, we found no significant effect of subsequent memory (*p* = 0.23), and no significant interaction between subsequent memory and stimulus category (*p* = 0.35). No other subfield showed increased fMRI signal change for remembered versus forgotten items.

Further, to show that scene-related activity was not greater in individuals slower to successfully discriminate scenes, we correlated interindividual variation in subicular scene BOLD response with individual mean RTs. We found no significant correlation between scene response and RT in the subiculum (*r* = 0.07, *p* = 0.77), or in any of the remaining hippocampal ROIs (all *p* values >0.5).

### Comparing anterior and posterior subfield regions

**Figure 3.**
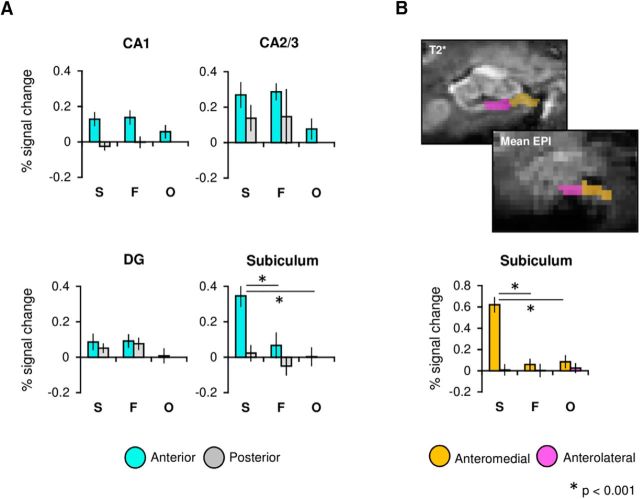
***A***, Percentage signal change plots for anterior and posterior subdivisions within each hippocampal subfield ROI (CA1, CA2/3, DG, and subiculum). ***B***, Example ROIs for the anteromedial and anterolateral subiculum shown on both the ultra-high-resolution T2* image and the mean EPI image of a single subject (top). Percentage signal change plot for the medial and lateral subdivisions of the anterior subiculum (bottom). Mean values (across subjects and task runs) are shown for scenes (S), faces (F), and objects (O). Error bars represent ± SE.

Recent work suggests that the anterior part of the hippocampus forms of a broader anterior scene-processing network that incorporates anterior parahippocampal place area (PPA), anterior retrosplenial cortex (RSC), and caudal inferior parietal lobule (cIPL; Baldassano et al., 2016). Further, it has been proposed that the anterior hippocampus, and in particular the subiculum, is critical for the formation of coherent scene representations during visual perception (Zeidman and Maguire, 2016). Based on this, we conducted an additional ROI analysis in which we subdivided each hippocampal subfield into anterior and posterior sections (see Materials and Methods). While we found a significant effect of category in the subiculum (*p* < 0.001), consistent with the analysis presented in the previous section, we also found a significant effect of long-axis region (*F*_(1,22)_ = 6.55, *p* = 0.018, η^2^_p_ = 0.23) and an interaction between these factors (*F*_(1,22)_ = 4.88, *p* = 0.016, η^2^_p_ = 0.18). The subicular response for each oddity condition differed in both the anterior (*F*_(2,44)_ = 12.63, *p* < 0.001, η^2^_p_ = 0.37) and posterior (*F*_(2,44)_ = 3.63, *p* = 0.038, η^2^_p_ = 0.14; Fig. 3*A*) subdivisions. In the anterior subiculum, the fMRI signal response during successful scene discriminations was significantly greater than both faces (*p* < 0.001) and objects (*p* < 0.001; Bonferroni-corrected α = 0.05/3 = 0.017), whereas no significant differences were found in the posterior subiculum following Bonferroni correction (scene vs face *p* = 0.23; scene vs object *p* = 0.03; face vs object *p* = 0.11). Further, only scene oddity judgements elicited significantly greater BOLD response in the anterior, relative to posterior, subiculum (*p* < 0.001; faces: *p* = 0.61; objects: *p* = 0.37). These results suggest, therefore, a potentially unique role for the anterior subiculum in perceptual scene discriminations.

Within our CA1 ROI, we found a significantly greater fMRI signal response in anterior versus posterior CA1 (*F*_(1,22)_ = 9.32, *p* < 0.001, η^2^_p_ = 0.3). While no significant main effect of stimulus category was found in CA1 (*p* = 0.56), a significant interaction between category and long-axis region was observed (*p* = 0.01). Unlike the subiculum ROI, follow-up tests revealed no significant effects of category in either anterior (*p* = 0.09) or posterior (*p* = 0.15) CA1. No significant main effect of category (*p* = 0.79) or long-axis region (*p* = 0.9) was observed in the DG, though an interaction was found between these factors (*p* = 0.01). Follow-up ANOVAs identified no significant differences between oddity categories in either the anterior (*p* = 0.08) or posterior (*p* = 0.37) DG. Direct comparisons between the anterior and posterior DG for each stimulus type likewise identified no significant differences (all *p* values >0.08). There were no significant effects in CA2/3 (all *p* values >0.11).

### Differences along the transverse axis of the subiculum

We next investigated the possibility that the scene effect in the anterior subiculum differed along its transverse axis. Previous group-level fMRI studies at 3 T have reported scene-selectivity in the anterior-*medial* region of the hippocampus (Zeidman et al., 2015a; Hodgetts et al., 2016), and electrophysiological studies in animals have revealed that place cells show greater spatial coherence and firing rate in the medial versus lateral subiculum (Sharp and Green, 1994; Kim et al., 2012). To test this potential functional distinction along the transverse axis, the anterior subiculum was divided into medial and lateral sections on individual structural scans (Fig. 3*B*). Notably, this analysis revealed a significant interaction between oddity condition and transverse region (*F*_(2,44)_ = 27.39, *p* < 0.001, η^2^_p_ = 0.56), whereby the response for the perceptual oddity conditions differed significantly in the medial (*p* < 0.001), but not lateral (*p* = 0.96), anterior subiculum (Fig. 3*B*). The scene response in the anteromedial subiculum was significantly greater than that shown for faces (*p* < 0.001) and objects (*p* < 0.001). Further, only scene discriminations elicited increased BOLD in the medial relative to lateral subiculum (*p* < 0.001).

### Differences in the retinotopic size of stimuli

To demonstrate that these between-category effects in the subiculum do not solely reflect differences in size, we compared the on-screen pixel area (i.e., retinotopic size) occupied by each oddity condition (scenes, faces, and objects). A significant difference between oddity conditions was observed (*F* = 1341.51, *p* < 0.001). While scenes were significantly larger than both faces (*p* < 0.001) and objects (*p* < 0.001), faces were also significantly larger than objects (*p* < 0.001). Thus, while scenes were the largest item category, face stimuli were also, on average, larger retinotopically than objects. Critically, therefore, these retinotopic size differences were not mirrored in terms of a similar numerical pattern in our main anteromedial subicular results. This result, alongside work showing hippocampal scene-sensitivity for size-matched items (Barense et al., 2010; Lee and Rudebeck, 2010; Hodgetts et al., 2016), indicates that these results are not driven by stimulus size.

### Group-level hippocampal activity during perceptual oddity

**Figure 4.**
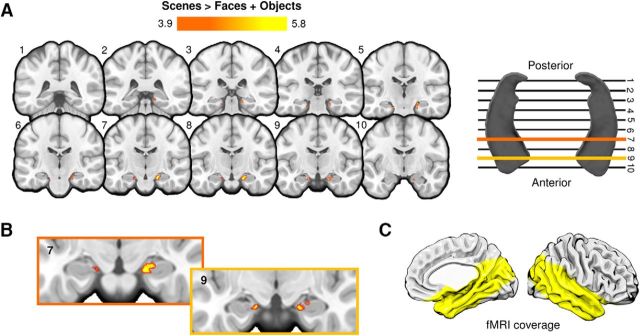
***A***, Significant scene oddity clusters within the hippocampal ROI. Clusters reflecting significantly greater activity for scenes > faces + objects are shown in red-yellow. The statistical map (FWHM, 2 mm) was thresholded at *p* = 0.0001 with a familywise error-corrected cluster threshold of *p* < 0.05. The coronal slices transect the long axis of the hippocampus, as shown in ***A*** (right). The slice intersecting the most posterior part of the hippocampal formation is depicted in slice 1, and the most anterior slice of the hippocampal body is shown in slice 10. ***B***, Magnified images of two slices (7 and 9) showing the location of scene-selective clusters in the anteromedial hippocampus. All activation maps are shown on the MNI152 standard template (1 mm). ***C***, Yellow highlighted region depicts the partial field-of-view coverage of our fMRI data.

To examine hippocampal activity at the voxel level across subjects, we conducted an additional group-level ROI analysis to test for significant increases in hippocampal activity during correct scene (vs face and object) oddity judgements (see Materials and Methods). For this analysis, probabilistic atlases of the hippocampus and subiculum were used to define a bilateral ROI of the hippocampus, and this was applied to the smoothed (FWHM, 2 mm), group-averaged functional data (see Materials and Methods). Group analyses (cluster-forming *p* = 0.0001, corrected) revealed significant bilateral activations in the hippocampus during scenes versus faces and objects (Table 2). The peak voxels within each cluster were in the region of the subiculum at the most medial point of the hippocampal formation (Fig. 4). Clusters were mostly constrained to the anterior hippocampus in both hemispheres.

**Table 2.** Hippocampal group-level activations for the perceptual scene oddity contrast

Scenes > Faces + Objects			*x*	*y*	*z*	Location	Hemisphere
Cluster #	Voxels	Max *Z*					
1	389	5.84	18	−17	−21	Subiculum/cornu ammonis	Right
2	107	5.19	−18	−25	−20	Subiculum	Left

The statistical map (FWHM, 2 mm) for scenes > faces + objects was thresholded at *p* = 0.0001 with a familywise error-corrected cluster threshold of *p* < 0.05. The location of cluster peaks in respect to individual hippocampal subfields is interrogated using the Jülich histological atlas (see Materials and Methods). Coordinates are in MNI152 space.

### Whole field-of-view activity during perceptual oddity

**Figure 5.**
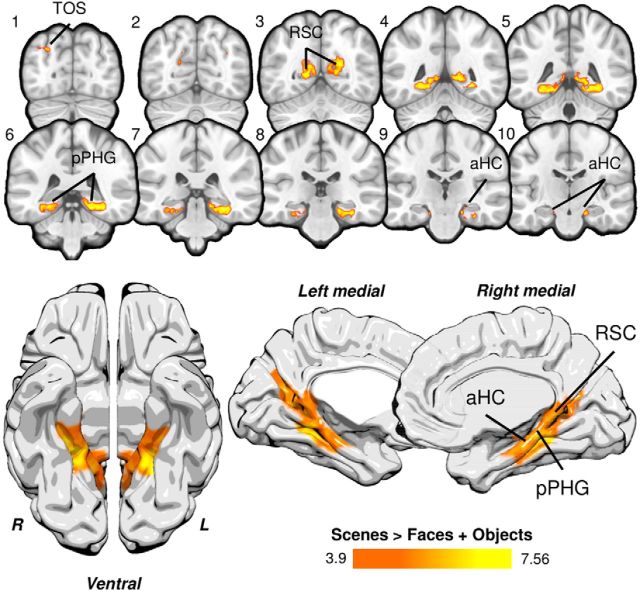
Significant whole field-of-view clusters for scene oddity. Clusters reflecting significantly greater activity for scenes > faces + objects are shown in red-yellow. Significant activity was found bilaterally in anterior hippocampus (aHC), posterior parahippocampal gyrus (pPHG), RSC, and lateral occipital cortex/transverse occipital sulcus (TOS). For visualization, the activation map was projected to the standard MNI152 template (top) and onto the ICBM152 brain template using Surf Ice software (bottom; https://www.nitrc.org/projects/surfice/). The statistical map (FWHM, 2 mm) was thresholded at *p* = 0.0001 with a familywise error-corrected cluster threshold of *p* < 0.05. The field-of-view for our fMRI data is shown in Figure 4*C*.

To explore category-selective activation outside our hippocampal ROI, an additional group analysis was conducted incorporating our whole functional field-of-view (*p* < 0.0001, corrected; Fig. 5). An analysis of correct scene (vs face and object) oddity trials revealed significant bilateral clusters incorporating the lingual gyrus, RSC, posterior parahippocampal gyrus, and hippocampal formation. Smaller clusters were found in the lateral occipital cortex/transverse occipital sulcus bilaterally. This broader network of regions during scene oddity corresponds to those regions showing strong intrinsic functional connectivity at rest (Baldassano et al., 2016), and increased functional activity during 3 T task fMRI (Hodgetts et al., 2016). The activation coordinates for this analysis are shown in Table 3.

**Table 3.** Whole field-of-view group-level activations for the perceptual scene oddity task

Scenes > Faces + Objects			*x*	*y*	*z*	Location	Hemisphere
Cluster #	Voxels	Max *Z*					
5	7947	6.83	28	−49	−6	Lingual gyrus	Right
4	5990	6.32	−22	−42	−13	Posterior PHG	Left
3	337	5.33	−30	−81	36	LOC/TOS	Left
2	73	4.94	37	−72	34	LOC/TOS	Right
1	41	4.83	41	−74	33	LOC/TOS	Right

The statistical map (FWHM, 2 mm) for scenes > faces + objects was thresholded at *p* = 0.0001 with a familywise error-corrected cluster threshold of *p* < 0.05. Coordinates are reported in MNI152 space.

LOC/TOS, Lateral occipital cortex/transverse occipital sulcus; PHG, parahippocampal gyrus.

### General discussion

Representational models suggest a potential key role of the hippocampus in the perceptual processing of complex visual scenes (Graham et al., 2010; Murray et al., 2016). Due to limitations at 3 T fMRI, however, it is currently unclear how this putative functional role maps onto the convoluted internal structure of the human hippocampus (Ding and Van Hoesen, 2015). To address this, we applied ultra-high-field 7 T fMRI, which allowed us to accurately delineate and localize the functional contribution of individual subfields during a perceptual discrimination task. A fine-grained ROI analysis of the non-normalized, unsmoothed fMRI data demonstrated increased activity during accurate perceptual scene discrimination (relative to other stimulus categories) in the subiculum but not CA1, CA2/3, or the DG. Additional analyses, based on segmentation of the long and transverse axes of this subfield, confirmed that this effect was in the anteromedial subiculum and was not modulated by whether perceptual targets were subsequently remembered or forgotten, thus highlighting a clear role for the anteromedial subiculum in viewpoint-independent perception of scenes.

These findings provide compelling support for accounts that propose an important role of the hippocampus in higher-order visual perception (Graham et al., 2010; Yonelinas, 2013), with the subicular subregion specifically showing a strong functional preference for scenes. One particular model suggests that the hippocampus is critical for the formation of complex, conjunctive scene representations (Bird and Burgess, 2008; Graham et al., 2010; Lee et al., 2012; Murray et al., 2016). Performance on tasks that place demand on these conjunctive representations, such as those involving the discrimination of highly overlapping visual scenes across multiple viewpoints (i.e., oddity), is impaired following hippocampal lesions (Lee et al., 2005a, 2006). Similarly, studies have demonstrated that hippocampal lesions impair the ability to discriminate scenes based on configural information (which involves binding of perceptual scene features), but not local details (Aly et al., 2013), which is consistent with spared performance on simple feature comparisons (Lee et al., 2005b; Mundy et al., 2013; e.g., shape, color, size). Alternative accounts emphasize a role for the hippocampus in the construction of internal scene models (Zeidman and Maguire, 2016). Indeed, studies have demonstrated impaired performance on tasks probing these constructive mechanisms, such as scene imagining (Mullally et al., 2012). Data regarding the role of specific hippocampal subfields in higher-order scene representation, however, have been elusive given limitations in anatomical resolution at 3 T. Using high-resolution fMRI in our study, we demonstrated that hippocampal contributions to scene processing may be better characterized as a unique and specific role of the subiculum subfield. Based on this new finding, we propose that the subiculum forms complex, viewpoint-invariant scene representations that may be used (in a task-directed manner) across both memory and higher-order visual perception, bolstering representational accounts (Graham et al., 2010; Murray et al., 2016). That this effect in the subiculum reflects perceptual processing, and not memory, is supported by (1) the minimal mnemonic demand of the task and (2) the finding that the subicular scene response was not modulated by subsequent memory performance. In particular, the application of concurrent stimulus presentation and trial-unique stimuli ensured that there was minimal requirement to maintain items in memory both within and across trials. Further, the time taken to successfully discriminate scene items, which could feasibly reflect short-term memory demands (Sternberg, 1969), was not associated with subicular BOLD response.

Not only do these findings place important constraints on theoretical models of hippocampal function, but may also resolve contradictions in the literature (Squire et al., 2006; Maguire and Hassabis, 2011; Kim et al., 2015). Critically, the development of more fine-grained, anatomically informed models of human hippocampal function using ultra-high-field MRI could help us understand the conditions under which perceptual impairments are observed, in addition to providing image resolution that allows differential subregional atrophy patterns to be quantified with greater precision in individuals with memory deficits (Wisse et al., 2014).

A critical role for the subiculum in higher-order scene processing is also suggested by work in animals. First, lesions to the subiculum in rats lead to comparable spatial learning and working memory deficits as seen for the hippocampus proper (Morris et al., 1990; Floresco et al., 1996; e.g., CA1–CA3 and the DG). Second, electrophysiological studies in rats have identified cells in the subiculum with distinct spatial firing properties, including place cells (Sharp, 2006), boundary vector cells (Lever et al., 2009), and cells attuned to the current axis of travel (Olson et al., 2017). Thus, the subiculum, by supporting the representation of location, orientation, and geometry, is well suited for the formation of complex viewpoint-invariant representations that may be used across memory and perception.

A second key finding was that our scene-selective effects were in the anterior region of the hippocampus. While several studies have observed scene-specific activations in the posterior hippocampus during perceptual discriminations (Lee et al., 2008, 2013; Barense et al., 2010; Zeidman et al., 2015a), others have demonstrated, using similar paradigms, involvement of the anterior subdivision (Lee et al., 2013; Zeidman et al., 2015a). A recent study, for instance, showed that the anterior hippocampus, relative to the posterior hippocampus, converges more strongly on a large-scale anterior scene processing network incorporating the anterior PPA, the anterior RSC, and the cIPL (Baldassano et al., 2016). The anterior hippocampus also responds strongly when the spatial configuration of scenes is altered (Howard et al., 2011) and during the passive viewing of scene exemplars (Zeidman et al., 2015a). A recent large-scale fMRI study confirmed robust group-level and individual-level scene activations in the anterior hippocampus during a one-back task (Hodgetts et al., 2016). The anterior hippocampus has been associated with global/coarse spatial representations, whereas the posterior hippocampus has been argued to support more fine-grained spatial processing (Poppenk et al., 2013). This has largely been informed by electrophysiological studies in rodents that have shown place cell receptive fields to increase in size from the posterior to the anterior hippocampus (but see Phillips and Eichenbaum, 1998; Kjelstrup et al., 2008). Recent computational models have indicated that larger place cells in the anterior hippocampus are not only equally precise, but may better subserve generalization across spatial locations (Keinath et al., 2014). The finding that anterior place cells represent larger, more overlapping areas of the environment suggests that anterior representations may be important in tasks requiring generalization across scene viewpoints, such as our scene oddity task.

We also found that our scene effect was in the medial versus lateral subiculum. This result converges with animal work showing that place cells in the medial versus lateral subiculum exhibit higher firing rates and are more strongly modulated by theta phase (Sharp and Green, 1994; Kim et al., 2012; Craig and McBain, 2015). A recent 3 T fMRI study observed increased BOLD response in the medial subicular region during scene recall (Zeidman et al., 2015b) but lacked the functional resolution applied here. Consistent with studies highlighting a role of the anterior hippocampus in a broader scene network (Baldassano et al., 2016; Hodgetts et al., 2016), a functional connectivity study at 7 T reported greater functional connectivity between the medial, relative to the lateral, subiculum and the posterior parahippocampal gyrus (Maass et al., 2015)—the latter a key scene-processing region (Marchette et al., 2015).

Here, through the application of high-resolution 7 T fMRI, we report scene sensitivity in the human hippocampus during a nonmnemonic, perceptual task. This finding provides compelling support for representational models of hippocampal function more broadly, but also refines this view by demonstrating that the putative role of the hippocampus in higher-order scene processing may be better characterized as a particular role of the anteromedial subiculum. The strong regional specificity reported here, afforded by ultra-high-field MRI, may help resolve inconsistencies in the literature and has implications for understanding neurological disorders that differentially affect specific hippocampal subregions. Alongside electrophysiological evidence from animals, these results speak broadly to the potential importance of subicular information processing in the formation of flexible scene representations that may underpin both spatial navigation and episodic memory.
